# Experimental Research on the Workability and Mechanical Properties of Grouting Materials with Nano-Oxides

**DOI:** 10.3390/ma16010329

**Published:** 2022-12-29

**Authors:** Honghong Zhou, Jun Liu, Yuanquan Yang, Runqing Liu

**Affiliations:** 1 School of Materials Science and Engineering, Shenyang Ligong University, Shenyang 110159, China; 2 School of Materials Science and Engineering, Shenyang Jianzhu University, Shenyang 110168, China

**Keywords:** nano-oxides, grouting materials, working properties, mechanical properties

## Abstract

This work studied the effect of the nano-oxides, such as Nano-Fe_2_O_3_ (NF), Nano-Al_2_O_3_ (NA), Nano-MgO (NM), and Nano-SiO_2_ (NS), on the workability and mechanical properties of quick-setting grouting materials serviced in the underground environment. The results show that 2.0% NS could remarkably shorten the setting time of the grouting materials by 29.16%, compared to the control one (without nano-oxides), and the final setting time was shortened by 46.51%. The results also show that 2.0% NS could decrease the initial fluidity of the grouting material by 41.09%, compared to the control one, and the 30 min fluidity was decreased by 48.93%. The XRD results show that NF, NM, and NS contribute to a higher quantity of AFt than that NA. Moreover, grouting material doped with NF, NM, and NS produces more needle-like ettringite, leading to a more compact structure.

## 1. Introduction

Concrete has been widely used in commercial buildings, water conservancies, tunnels, roads, and military buildings due to its advantages, such as good workability, excellent durability, convenient transportation, and low price [1]. However, cracks, bulging, and aggregate falling off could occur in the presence of harsh environments leading to the lower service life of concrete structures and potential dangers for quality and safety [2,3,4]. In construction, a large amount of concrete is used in the construction of underground projects, such as underground parking lots, tunnels, water conservancy projects, military air defense facilities, etc. [5,6]. For concrete structures and products serviced in underground engineering, the quality issues of concrete are attracting much concern due to humid and cold environments [7]. Some underground construction projects have quality problems and need to be repaired urgently, and some abandoned underground projects need to be repaired and utilized [8,9,10]. In the repair and maintenance of concrete products in underground engineering, many scholars have carried out research on repair materials, which has a positive significance for prolonging the service life and quality safety of construction projects [11,12,13]. At present, there are many kinds of repair materials for concrete used in underground construction projects, including organic, inorganic, and composite materials. Inorganic repair materials are widely used in the crack repair of concrete products due to their low price, good workability, and excellent durability.

As a kind of concrete material for crack repairing, inorganic grouting material has been widely used in concrete structures and products, and the technology is becoming more mature. In order to improve the workability and durability of grouting materials, many scholars have modified them with organic polyester, inorganic cementitious materials, or other materials, and achieved good results [7,14]. Nano-materials have very broad research prospects due to their special properties, such as high strength, high toughness, high magnetic susceptibility, and strong absorption rate of electromagnetic waves [15,16]. Research on nanomaterials-modified cement-based materials has become a research hotspot [17,18,19]. Khaldon Kasim Aswed enhances concrete performance by replacing part of the cement with nano-silica. The author found that NS can significantly improve the cement-aggregate interface transition zone by filling voids, depleting Ca(OH)_2_ crystalline particles, and generating more C-S-H gel, improving the mechanical and durability properties of concrete [20]. Chittaranjan B studied the hydration reaction process of ZnO and SiO_2_ nano-materials with C_3_S. Experimental results show that gel formed by nano-SiO_2_ is more helpful in improving the compressive strength and durability of concrete [21]. Reches Y et al. selected SiO_2_, TiO_2_, Al_2_O_3_, Fe_2_O_3_, bentonite, and halloysite nanoparticles (NPs) to study their agglomeration reaction in cement paste and their effects on compressive strength and thermal durability. The results show that TiO_2_, bentonite, and halloysite nano-particles enhanced the residual compressive strength of cement pastes exposed to high temperatures [22]. Wu Fufei selected several common nano-metal oxides such as Al_2_O_3_, CuO, ZrO_2_, MgO, Fe_3_O_4,_ and Fe_2_O_3_ to study their strong effects on the mechanical properties of cement-based materials. The results show that the nano-oxides can improve the flexural strength and compressive strength of cement-based materials, that the enhancement effect of nano-ZrO_2_ is the largest, and that of nano-CuO is the smallest [23].

Through the above research, it is found that the experimental results of various scholars have obtained different research results due to the difference in the selection of nano-oxides, material preparation methods, and experimental conditions. Many scholars mainly focus on the preparation of ordinary concrete and other products for the application of nano-oxides. On the preparation of grouting materials and their application in cold and humid underground environments, there are few records. In this study, the composition and content of metal oxides in cementitious materials such as sulphoaluminate cement, Portland cement, fly ash, and mineral powder were determined by XRF, and, instead of random selection, a relatively high-content metal oxides were selected. Considering the cost of nano-oxide, the effect of nano-oxides on grouting materials applied in damp and cold environments was studied using low content of nano-oxide.

## 2. Materials and Methods

### 2.1. Materials

In this test, the sulfoaluminate cement R·SAC 425 was produced by Liujiu Cement Co., Ltd. (Tangshan, China). Ordinary Portland cement P·O 525 was produced by Yangchun Cement Co., Ltd. (Zhucheng, China). Fly ash is grade II, produced by Guangmao Mineral Products Processing Factory, (Shijiazhuang, China). The mineral powder was produced by Shandong Xinruijie Chemical Co., Ltd. (Qingdao city, China). Quartz sand is produced by Hengxin Filter Material Factory, (Zhengzhou, China). Tap water was also used in this experiment. Through experimental detection, the main technical properties of cementitious materials and aggregates are shown in Table 1 and Table 2. The chemical composition of the cementitious material is shown in Table 3, Table 4 and Table 5.

In order to achieve good performance requirements of grouting materials, the following additives are used: (1) Defoamer, the main component is silicone oil, produced by Guanhao Chemical Co., Ltd. (Zhengzhou, China). (2) Water-reducing admixture, high-efficiency polycarboxylate water reducing admixture was produced by Chenqi Chemical Technology Co., Ltd. (Shanghai, China). (3) Retarder, industrial grade 99% sodium gluconate, which comes from Wolong Chemical Production, (Zhengzhou, China).

Nano-Fe_2_O_3_, Nano-Al_2_O_3_, Nano-MgO, and Nano-SiO_2_ are produced by Qinghe Kegong Metallurgical Materials Co., Ltd. (Xingtai, China). They’re all 50 nm in size; their densities are 2.12 g/cm^3^, 3.72 g/cm^3^, 3.60 g/cm^3^, and 2.40 g/cm^3,^ respectively. In the following studies, they are denoted by NF, NA, NM, and NS, respectively.

### 2.2. Test Methods

By adjusting the amount of cementitious material, admixture, and water, the fluidity, compressive strength, and flexural strength of the grouting material were tested to determine the mixing ratio and prepare the grouting materials. The NF, NA, NM, and NS were added to the grouting material with 0.4%, 0.8%, 1.2%, 1.6%, and 2.0%, respectively. When adding solid powder nano-oxides, continuous mechanical agitation is adopted to improve their dispersion. Referring to GB/T 1346-2011 [24], “Test methods for water requirement of normal consistency, setting time and soundness of the Portland cement”, the initial setting time and final setting time of the gelling material were measured using a Vicat instrument. The grouting materials are prepared with the same ratio, and the cement mortar mixer is used for mixing. After mixing, the grouting material is put into the circulating circular mold. According to GB/T 50448-2015 [25] “Technical code for application of cementitious grout”, the initial fluidity and 30 min fluidity of grouting material were tested. After the experiment, the grouting material was put into a 160 mm × 40 mm × 40 mm mold, solidified at 15 ± 1 °C and 90% humidity for 12 h, then released from the mold and solidified for 28 days. The compressive and flexural strength of grouting materials were measured referring to GB/T 17671-1999 [26] “Method of testing cements-determination of strength”. The air content and chord length frequency of the air pores in the dry grouting material were measured by rapid air equipment, and the test data were recorded. At the same time, the crushed sections of the samples were collected, put into anhydrous ethanol to stop hydration, then placed in a high-temperature drying oven at 80 °C for 8 h. Then the samples were put into a sealed bag. Some of the samples were ground to 200 mesh and the XRD test was carried out by the XRD-7000S (Cuka) X-ray diffractometer produced by Rigaku Corporation (Akishima-shi, Japan). The scanning speed is 0.06°~76.2°/min, and the angle measurement range is 5~45°. In addition, the broken test section was selected and broken into small parts of about 5 mm^3^, and the fresh section was coated with gold film and observed by using the S-4800 Scanning electron microscope made by Hitachi Company (Tokyo, Japan), (acceleration voltage of 10 kv).

## 3. Mix Ratio Design

The mix ratio design of grouting repair materials is designed according to the technical requirements in the two standards of GB/T 50448-2015 [25] “Technical code for application of cementitious grout” and JC/T 986-2018 [27] “Cement-based Grouting Materials”. The grouting material prepared in this study is quick-setting, and with the requirements for actual effect and early strength; therefore, the cementation material R·SAC 425 was selected. Adding a small amount of P·O525 into the cementitious material can compensate for its later strength shrinkage. Adding fly ash and mineral powder not only meets the performance requirements of grouting materials but also reduces the amount of cement, which reduces the cost. Because taking R·SAC 425 as the base material, the total amount of other cementitious materials, such as P·O 525, fly ash, and mineral powder, should not be greater than the total amount of R·SAC 425. Combined with the characteristics of cementified materials, the content of P·O 525 is determined to be 10% and 20%; fly ash content is 5%, 10%, 15%, and 20%; mineral powder content is 5%, 10%, 15%, and 20%. Quartz sand is chosen because it has higher strength than river sand, and the ratio of quartz sand to cementitious material is 1:1, which is more advantageous for recording and operating the experiment. According to the previous research and the mixing state and workability of grouting material, the water–cement ratio is set at 0.25, 0.26, 027, and 0.28. The water-reducing agent selects the high-efficiency polycarboxylate water-reducing admixture, which is commonly used in engineering. Its content is 0.3%, 0.35%, and 0.4%, which is based on the saturation point. The total mass of each group of cementitious materials was set at 900 g.

### 3.1. Amount of Cementitious Materials

The grouting material was prepared according to different content of R·SAC 425, P·O 525, fly ash and mineral powder, the 3d flexural strength, and 3d compressive strength of the grouting materials were tested. In the experiment, “3d” means that the grouting material is maintained in a specific environment for three days. According to the 3d strength test results of grouting materials with different amounts of cementitious materials, the cementitious material ratio with the maximum compressive strength was selected as the benchmark to prepare the grouting repair materials. When R·SAC 425 is 675 g, P·O 525 is 90 g, fly ash is 45 g, and mineral powder is 90 g, the compressive strength is the largest, which is 63.53 MPa, and the flexural strength is 8.016 MPa. Therefore, in the preparation of grouting repair materials, the content of P·O 525 is 10% of the total amount of cementitious materials, fly ash is 5% and mineral powder is 10%. Results of a 3d strength test of grouting materials with different amount of cementitious material are shown in Table 6.

### 3.2. Amount of Water

The amount of water in the grouting repair materials affects their fluidity and strength. The water-to-cement ratio is an important parameter in the design of the mix ratio. Through the technical index requirements of the fluidity and strength of the grouting materials, the water-cement ratio of the grouting materials was tested. The water–cement ratio of the grouting materials was set to 0.25, 0.26, 0.27, and 0.28. Since the addition of polycarboxylate water reducing admixture can affect the amount of water, the amount of polycarboxylate water-reducing admixture in the design of water–cement ratio was proposed 0.3%, 0.35%, and 0.4%. Results of workability test of grouting materials with different water cement ratio are shown in Table 7.

The initial fluidity, 3d flexural strength and compressive strength of grouting materials with different water–cement ratios and different amounts of superplasticizers were tested. In conjunction with national standards, Class III cement-based grout materials have a fluidity of no less than 290 mm. On the premise that the initial fluidity of grouting materials are not less than 300 mm, when the water-cement ratio is 0.25 and the content of polycarboxylate superplasticizer is 0.35%, the initial fluidity of grouting material is 306 mm, and the compressive strength is the highest, which is 62.24 MPa, and the flexural strength is 8.407 MPa. Therefore, the water–cement ratio of the proposed grouting material is 0.25, and the content of polycarboxylate superplasticizer is 0.35%.

### 3.3. Amount of Ad-Mixtures

Considering that sulfoaluminate cement has the characteristics of rapid setting, in order to ensure the construction time of grouting materials, sodium tetraborate and sodium gluconate, which have good retarding effect on the setting of sulphoaluminate cement, were selected as retarders for the test. The amount of retarder should be determined according to the standard requirement that the fluidity of Class III cement-based grouting materials should not be less than 260 mm in 30 min. Effect of retarder on fluidity of grouting materials are shown in Table 8.

The results of sodium tetraborate and sodium gluconate on the initial fluidity and 30 min fluidity of grouting materials were compared. Combined with the standard requirements of Class III cement-based grout material, when the initial fluidity is no less than 290 mm, and the 30 min fluidity is no less than 260 mm, the selection of sodium gluconate retarder can meet the fluidity requirements. When the dosage of sodium gluconate is 0.06%, the initial fluidity of the grouting material is 341 mm, and the 30 min fluidity is 282 mm. Therefore, in the preparation of grouting material, the selected retarder is sodium gluconate, and the dosage is 0.06%.

Because the grouting material produces a large number of pores during the stirring process, the result influences the strength and durability of the grouting material. In the test, silicone defoamer and special defoamer for polycarboxylate water reducing admixture (6018 defoamer, produced by Dongguan Meiyang Trading Co., Ltd. (Dongguan, China), and H500 defoamer, produced by Dachuan Fine Chemical Co., Ltd. (Guangzhou, China), were selected for experimental research. Effect of different defoamers on strength of grouting materials are shown in Table 9.

Comparing the effects of silicone defoamer, 6018 defoamers, and H500 on the 1d compressive strength and flexural strength of grouting materials, the strength of the grouting material mixed with silicone defoamer is better than 6018 and H500. When the content of silicone defoamer is 0.3%, the compressive strength is the largest, which is 53.71 MPa, and the flexural strength is high. So, the antifoaming agent uses silicone antifoaming agent, and the dosage is 0.3%.

Through the mixture–ratio test of the amount of cementitious material, water, and ad-mixture of grouting materials, the following results are obtained. The 3d flexural strength and 3d compressive strength of grouting material were tested according to the dosage of four kinds of cementitious materials. The optimum ratio of R·SAC 425, P·O 525, fly ash, and mineral powder was selected. The results show that the content of the cementitious material R·SAC 425 is 75% of the total amount of the cementitious material, P·O525 is 10%, the fly ash is 5%, and the mineral powder is 10%. The ratio of quartz sand and cementitious material is 1:1. The strength of the grouting materials with the water–cement ratio of 0.25, 0.26, 0.27, and 0.28, and the dosages of 0.3%, 0.35%, and 0.4% polycarboxylate water reducer were tested. The best group of three-day compressive strength is selected by the combination of the water–cement ratio and the amount of water-reducing agent, and then the water–cement ratio is 0.25, and the amount of polycarboxylate water-reducing agent is 0.35%. In addition, when the retarder is sodium gluconate, and the dosage is 0.06%, the initial fluidity and the 30-min fluidity can meet the requirements of GB/T 50448-2015 [25] “Technical code for application of cementitious grout”. When the defoamer is a silicone defoamer, and its content is 0.3%, the compressive strength of 1d is the highest. So the organosilicon defoamer with the content of 0.3% is selected. Based on the experimental results, the mixing ratio of grouting materials is shown in Table 10.

## 4. Results and Discussions

### 4.1. The Effect of Nano-Oxides on the Setting Time of Grouting Materials

**Figure 1:**
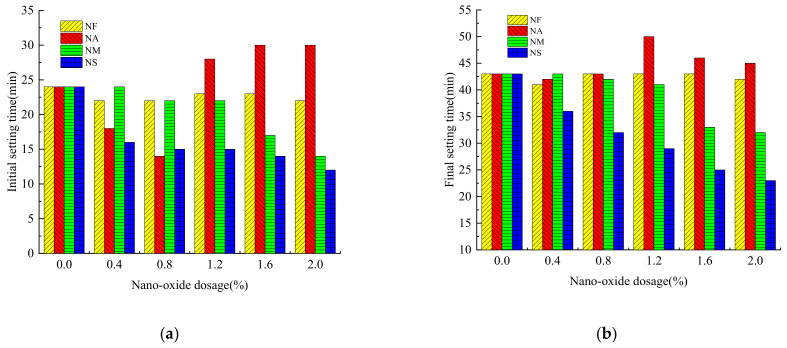
Effect of nano-oxides on setting time of grouting material. (**a**) Initial setting time; (**b**) Final setting time. The meanings of NF, NA, NM, and NS are Nano-Fe_2_O_3_, Nano-Al_2_O_3_, Nano-MgO, and Nano-SiO_2_, respectively.

Different types of nano-oxides and their dosages have different effects on the initial setting time and final setting time of the grouting material, as shown in Figure 1.

It can be seen from Figure 1 that the initial setting time and final setting time of the grouting cementitious material were changed with the addition of nano-oxides. As the content of NF increases, the initial setting time of the grouting material decreased compared to the grouting material without nano-oxides. While the initial setting time of the grouting material mixed with NA increases rapidly and then slows down with the increase of the content, and when the content is 0.8%, the initial setting time is the shortest, which is shortened by 41.67%. However, the initial setting time of the grouting materials mixed with NM and NS is gradually accelerated with the increase of the content, and the increase rate of NS is higher than that of NM, about 29.16% faster. The grouting materials doped with nano-oxides shorten the initial setting time. The NF-doped grouting materials have the smallest change in initial setting time, the largest change in NA, and the largest decrease in NS. For the final setting time, the effect of nano-oxides on the final setting time of the grouting material is similar to that of the initial setting time, with the smallest effect of NF and the largest reduction of NS. When the content of NS is 2.0%, it is shortened by 46.51% compared with the unmixed group, and the effect of NM is also greater. When the content of NS is 2.0%, the final setting time is shortened by 25.58%. However, when the content of NA is 1.2%, the final setting time is longer than other nano-oxides due to the phenomenon of “a false binder setting”.

In terms of the change of setting time, the influence of the incorporation of nano-oxides on the initial setting time and final setting time of the grouting material has changed, and most of the initial setting time and final setting time is shortened. For nano-oxides, because of their small particle size and large specific surface area, when they are incorporated into cement-based cementitious materials, more water is required to react with cement particles, so the amount of water in the hydration process of cement particles is reduced. It, in turn, shortens the setting time [28]. Moreover, due to the high chemical activity of nanomaterial, it can react with C_3_S in cement clinker to generate calcium carboaluminate, which accelerates the hydration rate of cement, thus reducing the setting time of grouting materials [29]. For NA, with the addition of its low content, the hydration process of sulfoaluminate cement particles can quickly participate in the hydration reaction and shorten the setting time. With the increase of NA content, the phenomenon of “a false binder setting” appeared, and the initial and final coagulation time showed a phenomenon of first shortening and then increasing [30]. Because NF cannot participate in the hydration reaction of cement, its incorporation has little effect on the setting time but can promote the hydration reaction of cement [31]. For the grouting material incorporating NM and NS, because both NM and NS have strong activity, their high surface activity requires more water, and can rapidly promote the hydration reaction of cement during the reaction process, so the setting time is equal to decreased rapidly [32]. For NS, the large specific surface area and pozzolanic activity are the main reasons that lead to the shortening of the setting time of grouting materials [33]. NS can hydrate with Ca(OH)_2_ produced by the hydration of cement and promote the hydration reaction to shorten the setting time of cement slurry [34,35].

### 4.2. Influence of Nano-Oxides on Fluidity of Grouting Materials

**Figure 2:**
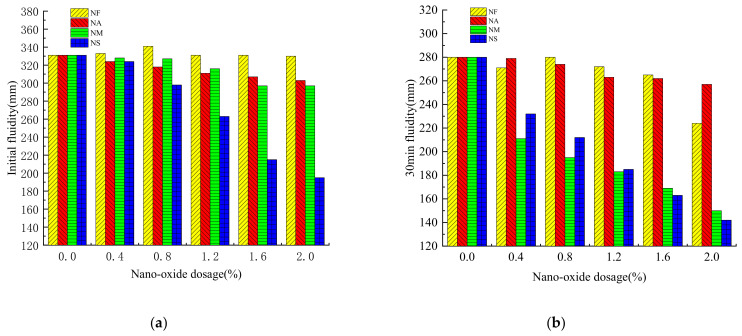
Effect of nano-oxides on fluidity of grouting materials. (**a**) Initial fluidity; (**b**) 30 min fluidity. The meanings of NF, NA, NM, and NS are Nano-Fe_2_O_3_, Nano-Al_2_O_3_, Nano-MgO, and Nano-SiO_2_, respectively.

The fluidity can evaluate the workability of the grouting material, and can ensure sufficient operating time in the actual construction. The fluidity of the grouting material mixed with nano-oxides shows different trends with the type and dosage of nano-oxides (Figure 2).

It can be seen from Figure 2 that the initial fluidity and a 30 min fluidity of the grouting material doped with nano-oxides show a decreasing trend with the increase of the content of nano-oxides. NS has the greatest influence on the initial fluidity of grouting materials, and its influence decreases by 41.09% when the dosage is 2.0%. With the addition of NF, NA, and NM, the initial fluidity also decreased with the increase of nano-oxides content, but the decrease was not as large as that of NS. The initial fluidity of the grouting material mixed with NF has the smallest decrease, which is only 0.30%, followed by NA, which is 8.46%, and NM is slightly larger, which is 10.27%. When the content of NM was 1.6%, the initial fluidity decreased by 10.27%. For the 30 min fluidity of the grouting material, it shows a decreasing trend with the incorporation of nano-oxides. It can be clearly seen from Figure 2 that the 30 min fluidity of grouting materials doped with NM and NS have the largest decreases, which are 46.43% and 48.93%, respectively. When the dosage is 0–1.2%, the 30 min fluidity of the grouting material doped with NM decreases more than that with NS. While the dosage is 1.2–2.0%, the reduction of NS is larger. The fluidity of the grouting materials doped with NF and NA decreases slightly, which is 20% and 8.21%, respectively. The fluidity of NA-doped grouting material at 30 min decreases gradually, which is different from the trend of NF-doped grouting material.

There are several reasons for the influence of nano-oxides on the fluidity of grouting material. With the incorporation of nano-oxide particles, the pores between the cement particles are filled, thereby draining the water in the pores, reducing the “lubrication” effect, and making the slurry denser. This increases the “resistance” between particles, resulting in a decrease in the fluidity of the grouting material [31]. Moreover, the nano-oxide has many fracture bonds in the preparation process, which make its surface energy high. When it is mixed into the grouting material, more water is needed to react with the cement particles. Furthermore, the adsorption phenomenon of nano oxides on cement particles will form a flocculent structure slurry to a certain extent. Combined with the thickening effect brought by the surface water absorption of nano oxides, the fluidity of grouting materials will be reduced [36]. The more the content of nano-oxides, the stronger the activity, the higher the viscosity of the grouting material, and the lower the fluidity [28]. For the initial fluidity, the fluidity of grouting material mixed with NS decreases most obviously, which indicates that NS has a large specific surface area, which increases the water requirement of grouting material [35]. For the 30 min fluidity, compared with the grouting materials with NF and NA, because NM and NS have very strong activity, they can promote the hydration reaction of cement rapidly when they are added into the grouting material, so the phenomenon of rapid setting appears, resulting in a rapid decline in fluidity [32]. Moreover, the activity of NF and NA was much lower than that of NM and NS, which could promote the hydration reaction, but the degree of reduction was relatively low.

### 4.3. Influence of Nano-Oxides on the Strength of Grouting Materials

**Figure 3:**
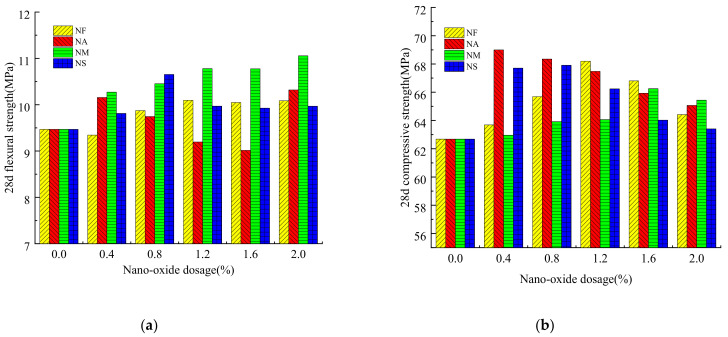
Effect of nano-oxides on 28d strength of grouting material. (**a**) 28d flexural strength; (**b**) 28d compressive strength. The meanings of NF, NA, NM, and NS are Nano-Fe_2_O_3_, Nano-Al_2_O_3_, Nano-MgO, and Nano-SiO_2_, respectively.

The compressive strength and flexural strength of grouting materials mixed with different nano-oxides also show different influence laws. The details are shown in Figure 3.

It can be seen from Figure 3 that the 28-day strength of the grouting materials changes with the nano-oxides content. The 28d flexural strength of the grouting material mixed with NM showed a gradually increasing trend. The grouting material mixed with NF also showed a gradually increasing trend when the content was greater than 0.8%. The flexural strength of the grouting material mixed with NS is the highest when the content is 0.8%. For the grouting material mixed with NA, when the content of NA is 0.4–1.6%, the 28d flexural strength gradually decreases, and the strength is optimal when the content of NA is 2.0%. For the 28d compressive strength, the grouting material doped with NF is the densest and of the highest strength when the dosage is 1.2%, which is 8.79% higher than that of the grouting material without doped nano-oxides. When the content of NM was 1.6%, the 28d compressive strength of the grouting material increased by 5.69%; the strength of NS mixed with 0.8% increased by 8.34%, and the strength of NA mixed with 0.4% was 69.00 MPa, an increase of 10.08%.

By analyzing the 28d strength of the grouting material, it is concluded that the strength of the grouting material mixed with nano-oxides increases with the increase of the curing age. The flexural strength and compressive strength of the grouting material mixed with NF have increased, and both have the maximum strength when the dosage is 1.2%. Because NF has a large surface area and strong reactivity, it can promote the hydration reaction of cement and fill in-between cement hydration products to improve their compactness. When the grouting material mixed with NA is 0.4%, it can promote the hydration reaction of cement and exert the nano-effect. However, with the increase of the content of NA, the formed gel product affects the strength of the grouting material. Therefore, when the content of NA is 0.4–1.6%, the compressive strength of 28d shows a decreasing trend with the increase of the NA content. However, when the dosage is 2.0%, due to the large surface area and strong surface activity of NA, the hydration reaction requires more water; the water–cement ratio of the grouting material decreases sharply, and the effect of the water–cement ratio is greater than the effect of NA, so it shows that the compressive strength increases when the dosage is 2.0%. The strength development trend of the grouting material mixed with NM is similar to that of NF. The 28d flexural strength increases with the content increase, and the compressive strength reaches the maximum when the content is 1.6%. In addition to the high surface area and activity of NS, the NS can also participate in the hydration reaction of cement, consume calcium hydroxide in the cement body, make it generate C-S-H gel, and promote the hydration-reaction process of cement. All four nano-oxides can improve the strength of grouting materials to a certain extent [28]. In the process of cement hydration and hardening, a certain number of pores formed and the incorporation of nano-scale oxides into the grouting material can reduce the pores of the cement-based material to a certain extent and achieve the purpose of refining its pore size. Therefore, after nano-materials are incorporated into cement-based materials, the effective area of the cement-based materials under stress can be increased, thereby improving their mechanical properties. In the hydration process of sulfoaluminate cement, the main products of hydration are calcium sulfoaluminate hydrate (AFt and AFm), calcium silicate hydrate gel (C-S-H gel), aluminum glue, iron glue, and gel glue holes, etc. Nano-oxides have relatively fine particles and high activity. When incorporated into cement-based materials, they will promote the hydration of cement materials. The quantity of hydration products is relatively large, and they are intertwined with each other to improve mechanical properties [37]. Furthermore, it may be that the nano-oxide can adsorb Ca(OH)_2_ in the system and promote the growth of Ca(OH)_2_ on its surface, thus reducing the system’s alkalinity. The AFt produced at low basicity has less expansion, which results in the reduction of micro-cracks due to expansion in the hardened paste. Therefore, the strength of the grouting materials is higher than that of the non-doped nano-oxides [38].

### 4.4. Influence of Nano-Oxides on the Pores of Grouting Materials

#### 4.4.1. Pores Structure Analysis of Different Nano-Oxides Grouting Materials

**Figure 4:**
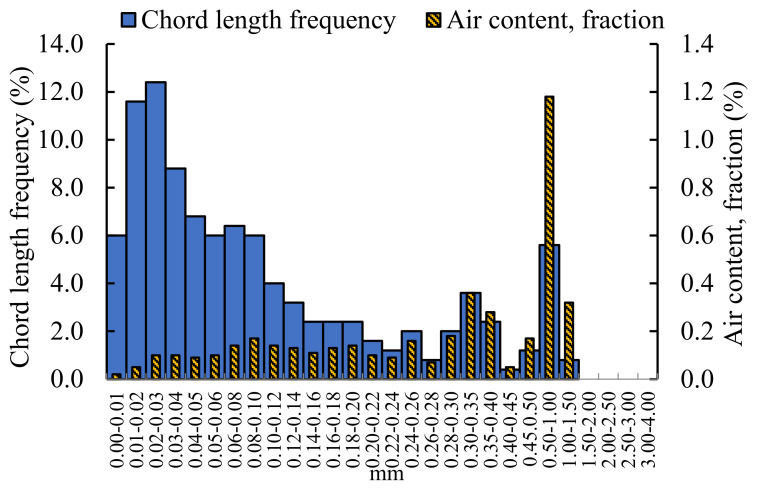
Analysis of pores structure of grouting material without nano-oxides.

The grouting materials with the best compressive strength of 28d doped with nano-oxides were selected. After cutting, grinding, and cutting surface treatment, the rapid air equipment was used to analyze the internal pores structure of grouting materials doped with different nano-oxides. The Rapid air instrument, which automatically analyzes the pore structure of hardened concrete, is produced by CXI company (Vedbaek, Denmark). It is used to automatically analyze the air content of hardened concrete with a microscope to measure the pore distribution. By analyzing the percentage of air content of pores in the grouting material, the content of a certain pores size in the grouting material can be determined. By analyzing the chord length frequency, the size of the internal pores can be determined. Therefore, by analyzing the percentage of air content and the chord frequency of the grouting material, the influence of different types of nano-oxides on the performance of the grouting materials was judged. The analysis of pores structure of grouting material without nano-oxides is shown in Figure 4.

The pores structure of the undoped nano-oxides grouting materials can be seen in Figure 4. The pores diameter of the grouting material is mainly distributed between 0.01 mm–1.50 mm. The air content of pores with a diameter of 0–0.5 mm is evenly distributed. The air content of pores with a pore diameter of 0.50–1.00 mm is the largest, accounting for 1.18% of the total air content, and no pore diameter exceeding 1.50 mm is found. From the perspective of chord length frequency, the chord length is mainly concentrated below 0.10 mm, the largest chord length frequency is 0.02–0.03 mm, accounting for 12.4%, followed by 0.01–0.02 mm, and the chord length frequency percentage is 11.6%. According to the air content and chord length frequency, it can be concluded that the pores without nano-oxides grouting materials are mainly composed of pore diameters below 1.50 mm and are uniformly distributed.

**Figure 5:**
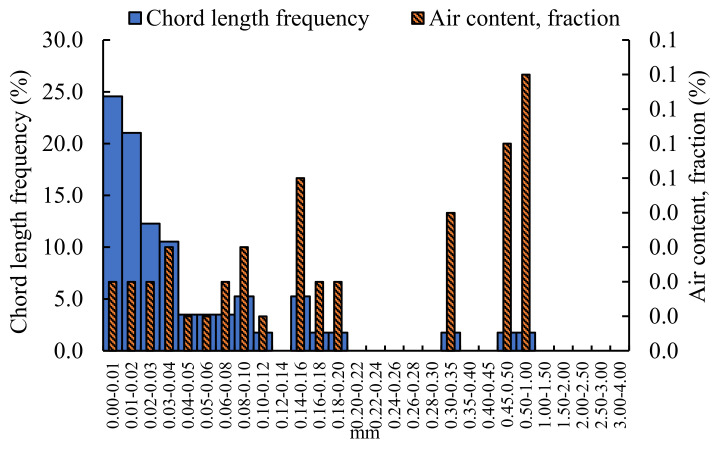
Analysis of pores structure of grouting material mixed with 1.2% NF.

The pores structure of grouting material mixed with 1.2% NF is analyzed, and the results are shown in Figure 5.

The diameter of the pores in the grouting material mixed with NF is mainly concentrated in intervals of 0–0.20 mm, 0.30–0.35 mm, and 0.45–1.00 mm. The air content of pores with a pore diameter of 0.50–1.00 mm is the largest, which is 0.08%, followed by the pore diameter range of 0.45–0.05 mm, and the air content is 0.06%. From the perspective of chord-length frequency, it is mainly concentrated in a diameter of 0–0.04 mm; the chord-length frequency of the diameter of the pores below 0.01 is the largest, which is 24.56%, followed by the pore diameter of 0.01–0.02 mm, which is 21.05%. It can be concluded that the grouting material mixed with NF has only small pores and few pores.

**Figure 6:**
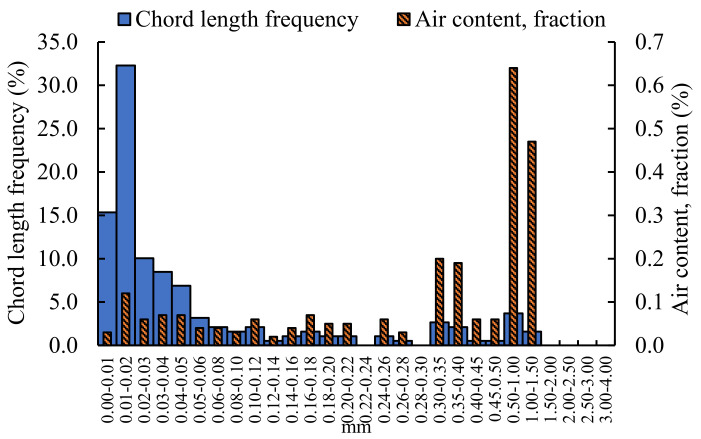
Analysis of pores structure of grouting material mixed with 0.4% NA.

The pores structure of grouting material mixed with 0.4% NA is analyzed, and the results are shown in Figure 6.

As can be seen from Figure 6, the size of the pores mixed with 0.4% NA grouting materials ranges from 0.00 to 1.50 mm, and the air content is the largest in the size of 0.50–1.00 mm, which is 0.64%, followed by 1.00–1.50 mm, which is 0.60%. From the chord length frequency, the pore size is the largest in the area of 0.01–0.02 mm, accounting for 32.28%, and the chord frequency in the 0.02–0.05 mm aperture range is slightly higher than other aperture ranges. It can be concluded that there are individual large pores in the pores structure mixed with NA grouting material, and the micro-dense pores are still the main ones.

**Figure 7:**
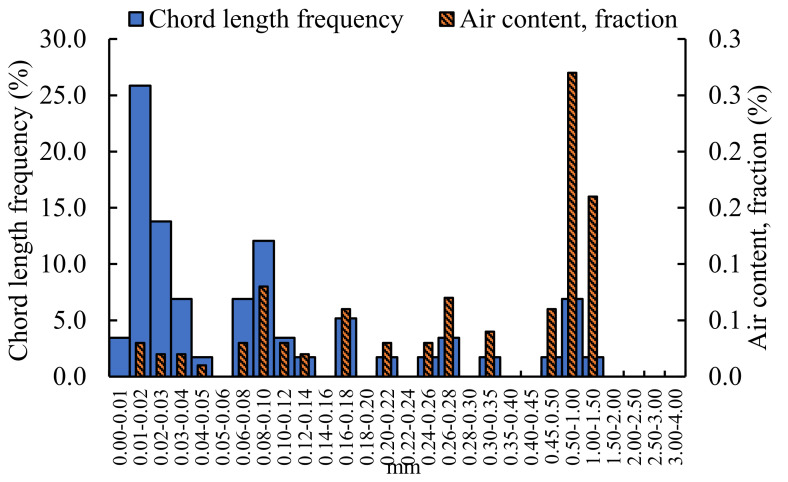
Analysis of pores structure of grouting material mixed with 1.6% NM.

The pores structure of grouting material mixed with 1.6% NM is analyzed, and the results are shown in Figure 7.

Figure 7 also presents that the air content is the largest for the grouting material mixed with 1.6% of NM. For example, the air content accounted for 0.27% of the pores with a diameter of 0.5–1 mm, and 0.16% of the pores with a diameter of 1–1.5 mm. The chord frequency is mainly concentrated in the two regions of 0.01–0.04 mm and 0.06–0.10 mm, and the chord frequency in the 0.01–0.02 mm region is much more than other regions, accounting for 25.86%. Overall, the air content is less than that of the undoped nano-oxides, and the size of the pores is smaller.

**Figure 8:**
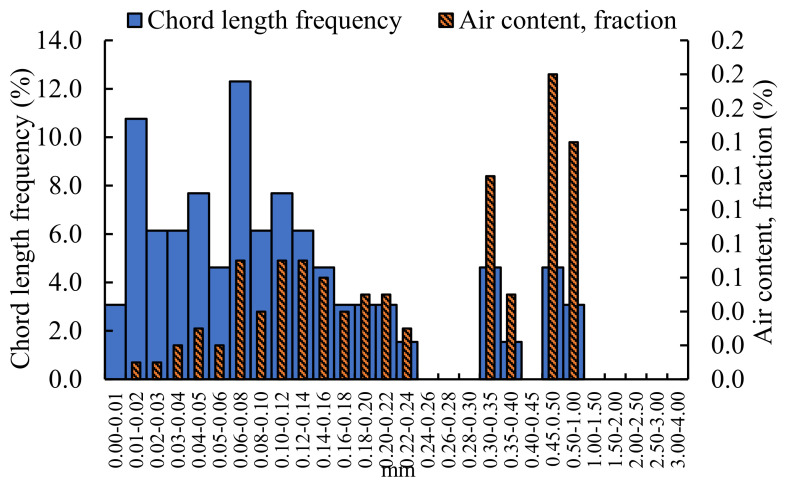
Analysis of pores structure of grouting material mixed with 0.8% NS.

The pores structure of grouting material mixed with 0.8% NS is analyzed, and the results are shown in Figure 8.

The results for the pore structure of the grouting material mixed with 0.8% NS show that when the pore diameter is 0.45–0.50 mm, the air content is the largest, which is 0.18%. When the aperture is 0.50–1.00 mm and 0.3–0.35 mm, the air content is 0.14% and 0.12%, respectively. Some pores are mainly distributed in the pore diameter of 0.01–0.24 mm. The chord length frequency also presented that as the pores aperture is 0.06–0.08 mm, the chord length frequency is the largest, which is 12.31%, and the chord length frequency is mainly concentrated below the 0.16 mm aperture. It shows that the grouting material with NS is dense in structure and less in air content.

Comparing the air content and chord frequency of the unincorporated nano-oxides grouting materials with that doped nano-oxides grouting materials, the size of the pores without nano-oxides grouting materials are mainly distributed below 1.50 mm, and areas are densely and uniformly distributed. The diameter of pores mixed with NF grouting materials are mainly concentrated in three areas: 0–0.20 mm, 0.3–0.35 mm, and 0.45–1.00 mm. In contrast, the air content with NA between 0.5–1.50 mm pores size are the most, and other areas are dominated by dense small pores. After incorporating NM, the air content is still concentrated in the area of 0.5–1.50 mm, but compared with the grouting material incorporating NA, the content is less. The air content mixed with NS is concentrated in the 1.00–1.50 mm region, and there are more small-pore size pores. The air content of the grouting materials doped with nano-oxides are less than that of the grouting material not doped with nano-oxides. The air content of undoped nano-oxides grouting material reached a maximum of 1.18%, followed by NA-doped grouting material, which was 0.64%, and the air content of other nano-oxide-doped grouting materials was smaller, all below 0.3%. From the perspective of chord frequency, the chord frequency of undoped nano-oxides is similar to that of doped NS grouting material, and is concentrated below 0.20 mm. For the grouting materials mixed with NF, NA, and NM, most of the chord frequencies are concentrated in the aperture range of 0–0.02 mm, and other chord frequencies are distributed in a staggered manner.

#### 4.4.2. Analysis of the Pore-Scanning Structure of Different Nano-Oxides Grouting Materials

**Figure 9:**
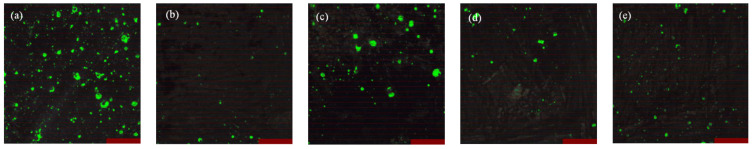
Scanning diagram of pore structure of grouting material. (**a**) Undoped; (**b**) 1.2% NF; (**c**) 0.4% NA; (**d**) 1.6% NM; (**e**) 0.8% NS.

The internal cutting surface of the grouting materials was scanned and photographed by rapid air equipment; the cross-section was scanned, and 800 pictures were obtained. The small images were then combined into a stomatal scanning structure picture of the cross-section of the grouting material. In the picture, the black part is the grouting material, and the green part is the pores of different sizes and structures. By analyzing the content, size, and shape of air pores in the cross-section scan of the grouting material, the influence of nano-oxides on the grouting material was further clarified. Figure 9 shows the scanning diagrams of pore structures of grouting materials without nano-oxide and with 1.2% NF, 0.4% NA, 1.6% NM and 0.8% NS.

Figure 9 presents that the pores structure without nano-oxides grouting material gives more pores than that with nano-oxides, and the distribution is more uniform. However, after the nano-oxides are doped, the number of pores is much less than that of the undoped nano-oxides, and they are all small pores in structure. In the grouting material doped with NF, the pores structure is dominated by small pores, and the number of pores is much smaller than that of the grouting material without nano-oxides, and most of the pores are mainly circular. Compared with the grouting material without nano-oxides, the number of pores in the grouting material doped with NA is less, and most of the pores are of a round structure. The pores structure of the grouting material mixed with NM is similar to that of NS, but the number of pores in the NS-doped grouting material is slightly higher than that of the NM-doped material, and the pores are densely distributed. The pores doped with nano-oxide grouting material have the largest number of NA-doped, the least number of NF-doped, and the most uniform distribution of NS-doped.

After the nano-oxide is added to the grouting materials, the high activity of nano-oxides can rapidly reduce the amount of water used in the grouting materials, resulting in a decrease in the number of pores brought in during the stirring process. In the pore structure of the grouting materials, the air content and number of large pores are reduced [32]. Because of the filling effect of nano-oxides, the nano-oxides can fill in the micro-pores of grouting materials, and the addition of nano-oxides can promote the formation of more C-S-H gel. Under the influence of nano-induced hydration, the gel becomes more compact [39]. The filling effect of nano-materials and the hydration of grouting materials promoted by nano-materials can optimize the microstructure of grouting materials [19]. Therefore, in the pore structure of the grouting materials, the air content of small pores and tiny pores is much lower than that of the grouting materials without nano-oxides.

### 4.5. XRD Curves of Grouting Materials with Different Nano-Oxides

**Figure 10:**
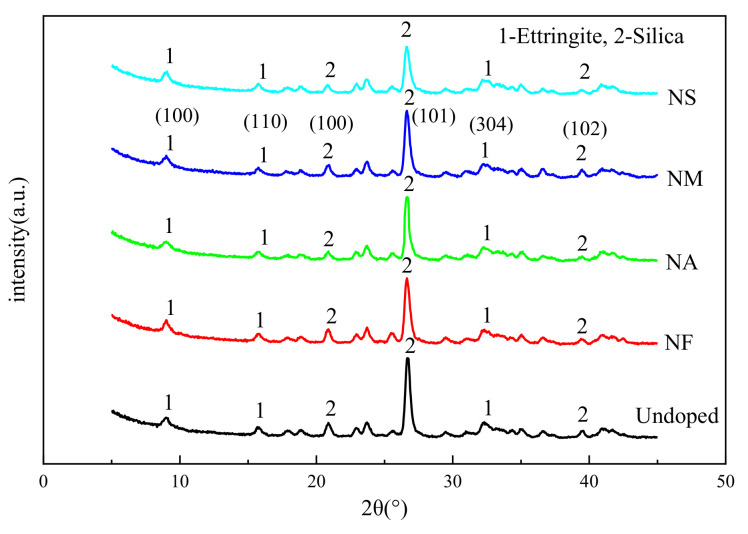
XRD curves of grouting materials with different nano-oxides.

In order to study the effect of incorporating different nano-oxides on the grouting materials, the grouting materials doped with nano-oxides were selected for XRD. The age of the grouting materials were 28 days, and the results are shown in Figure 10.

It can be seen from Figure 10 that the composition of the hydration products of the grouting material without nano-oxides is basically similar to that of the grouting materials doped with NF, NA, NM, and NS. The cement clinker of sulfoaluminate cement (SAC) mainly includes anhydrous calcium sulfoaluminate (C_4_AS), dicalcium silicate (C_2_S), and gypsum (CaSO_4_ 2H_2_O), etc. The main hydration products are calcium sulfoaluminate hydrate (AFt and AFm), calcium silicate hydrate gel (C-S-H), aluminum glue and iron glue, etc. The cementitious material in the grouting material is mainly sulfoaluminate cement, and the hydration reaction is rapid. Since gel-like products could not be detected in XRD, the degree of hydration reaction was mainly judged by hydration products AFt and AFm. In this study, the content of nano-oxides was low, which had little effect on the hydration process. In addition, the curing environment of grouting materials is non-standard, with an ambient temperature of 15℃ and humidity of 90%, which affects the hydration reaction of sulphoaluminate cement. Therefore, the diffraction peak intensities of the resulting calcium sulfoaluminate hydrate are slightly different. It can be seen from XRD that when 2θ are about 9.08°, 15.8°, and 32.26°, respectively, the AFt diffraction peak is stronger, and the interplanar distances are 9.72, 5.61, and 2.77, respectively. From the diffraction peak intensity of doped nano-oxides, the diffraction peaks of NF, NM, and NS are stronger than those of NA, indicating that the incorporation of NF, NM, and NS can promote the hydration of sulfoaluminate cement more than the incorporation of NA.

After incorporating NF, no iron oxide was found in the hydration product of the grouting material, but AFt with Fe_2_O_3_ was detected. It shows that the alumina in the hydrated calcium sulfoaluminate is replaced by the ferric oxide; the hydrated product still exists in the form of AFt and has high strength. The hydration product of the grouting material mixed with NA is mainly calcium sulfoaluminate hydrate. After incorporating NA, the hydration product AFt of the grouting material is less than that of others. Since Ca(OH)_2_ was not found in the hydration product, it can be inferred that a part of NA participated in the hydration reaction to form calcium aluminate hydrate (C-A-H) gel. After incorporating NM, it can promote the hydration reaction and generate ettringite (Aft), which can improve the strength of the grouting material. Part of NM can also react with the hydration product Ca(OH)_2_ to form hydrated magnesium silicate (M-S-H) gel. The incorporated NS can also react with Ca(OH)_2_ in the hydration product to generate calcium silicate hydrate (C-S-H) gel, and at the same time, due to the consumption of Ca(OH)_2_, the concentration of Ca(OH)_2_ is reduced, which in turn, accelerates the hydration reaction of cement, making the reaction fast [40,41].

### 4.6. SEM Microstructure of Different Nano-Oxides Grouting Materials

**Figure 11:**
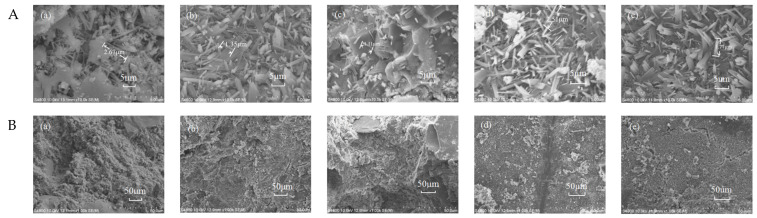
SEM image of grouting. (**A**): Grouting materials amplified to 5 μm: (**a**) Undoped; (**b**) 1.2% NF; (**c**) 0.4% NA; (**d**) 1.2% NM; (**e**) 0.8% NS. (**B**): Grouting materials amplified to 50 μm: (**a**) Undoped; (**b**) 1.2% NF; (**c**) 0.4% NA; (**d**) 1.2% NM; (**e**) 0.8% NS.

In SEM, the grouting material with the highest compressive strength among different nano-oxides is selected, and the influence of nano-oxide on the grouting materials was analyzed through the microscopic morphology and the degree of compaction, as shown in Figure 11.

It can be seen that the density of the grouting materials is higher than that without the addition of nano-oxides, indicating that the nano-oxides can enter the pores of the hydration product and increase the compaction effect. Because nano-oxides in the grouting materials can disperse cement particles, increase the contact area between cement and water, and then can be used as the nucleation base of C-S-H gel. Due to their adsorption, the concentration of hydration products around the nano-particles can be reduced, and the purpose of accelerating cement hydration and increasing the crystal nuclei. Moreover, when the dosage of nano-materials is appropriate, the generated C-S-H gels are intertwined, which also increases the compactness of the hydration product to a certain extent. In the grouting materials without nano-oxides incorporation, it can be seen that the needle-like ettringite and the amorphous hydrated calcium silicate gel are intertwined. After incorporating NF, a large amount of acicular ettringite exists in the hydration product of the grouting material, which improves the mechanical properties of the grouting material. Although NF is active and contributes to the formation of ettringite, it cannot take part in the secondary hydration of sulphoaluminate cement. NF plays an important role in filling and compacting the grouting material, refining the pore structure of the grouting material and promoting the hydration process of SAC [41]. After incorporating NA, the resulting ettringite and the hydration gel product structure are intertwined, and a small amount of white fine particles are adsorbed on the surface of the hydration product, which may be the AFm structure transformed from AFt. Moreover, NA can participate in secondary hydration to generate C-A-H, C-A-S-H and C-S-H gels, which further improve the compactness of grouting materials [42]. In the hydration products doped with NM, ettringite structure is denser and more staggered and elongated acicular calcium-vanadium structure is denser, which is also the result of the hydration promoted by the addition of NM. However, the structure of ettringite is short rod structure after adding NS, because NS is easier to react with Ca(OH)_2_ to form C-S-H gel, so the hydration reaction rate is faster and the alkaline environment is changed, the structure of ettringite also changed [43].

## 5. Conclusions

(1) Nano-oxides can shorten the setting time and fluidity of grouting materials. The initial setting time of the grouting material mixed with NA increases rapidly and then slows down with the increase of the content with a content of 0.8%, the initial setting time is the shortest, and the setting time is shortened by 41.67%. For the final setting time, the reduction degree of NS was the largest, and when the dosage of NS was 2.0%, the final setting time was shortened by 46.51% compared with the unmixed group. Among the influences of initial fluidity, NS has the greatest influence on the initial fluidity of grouting materials. When the dosage is 2.0%, it decreases by 41.09%, while the 30 min fluidity of grouting materials doped with NM and NS has the greatest decrease, each of which is 46.43%, 48.93%.
(2) Incorporation to different degrees. As NF, NA, NM and NS content were 1.2%, 0.4%, 1.6% and 0.8%, the compressive strength of the grouting materials presented excellent.
(3) The air content of the grouting materials doped with nano-oxides is smaller than that of the grouting material not doped with nano-oxides. The chord frequency of the grouting material mixed with NS is concentrated below the length of 0.20 mm, and the chord frequency of the grouting material mixed with NF, NA and NM is mostly concentrated in the range of 0–0.02 mm. The pores of the non-doped nano-oxides grouting material are more than that of the nano-oxides-doped. After doped with nano-oxides, the number of pores appearing is much less than that of undoped nano-oxides, and they are all small pores in structure.
(4) The hydration products of the grouting material without nano-oxides is basically similar to that of the grouting materials doped with NF, NA, NM, and NS. The grouting materials incorporating NF, NM, and NS can generate more AFt, thereby improving their durability.
(5) In a reasonable range of content, four kinds of nano-oxides (NF, NA, NM, NS) can be used in sulphoaluminate cement-based grouting materials.

## Figures and Tables

**Table 1:** The main technical properties of cementitious materials.

Class	Type	Density	Fineness	Stability
R·SAC 425	------	2.86 g/cm^3^	qualified	qualified
P·O 525	------	3.15 g/cm^3^	qualified	qualified
Fly ash	Ⅱ	2.42 g/cm^3^	------	------
Mineral powder	S95	2.86 g/cm^3^	------	------

**Table 2:** The main technical properties of quartz sand.

Class	Bulk Density	Apparent Density	Fineness Modulus
Quartz sand	1510 kg/m^3^	2.4 g/cm^3^	2.4

**Table 3:** The chemical composition of cement.

Class	CaO (%)	Al_2_O_3_ (%)	SO_3_ (%)	SiO_2_ (%)	MgO (%)	Fe_2_O_3_ (%)	TiO_2_ (%)	K_2_O (%)	Na_2_O (%)	Loss (%)
R·SAC425	42.22	24.92	15.13	10.55	3.00	1.76	0.92	0.63	0.50	0.36
P·O525	55.64	8.24	3.41	22.72	3.40	3.37	0.41	1.36	1.10	0.31

**Table 4:** The chemical composition of fly ash.

SiO_2_ (%)	Al_2_O_3_ (%)	Fe_2_O_3_ (%)	CaO (%)	TiO_2_ (%)	K_2_O (%)	MgO (%)	SO_3_ (%)	Na_2_O (%)	Loss
48.32	37.53	5.45	4.32	1.2	1.11	0.8	0.76	0.3	0.19

**Table 5:** The chemical composition of mineral powder.

CaO (%)	SiO_2_ (%)	Al_2_O_3_ (%)	MgO (%)	SO_3_ (%)	TiO_2_ (%)	Na_2_O (%)	K_2_O (%)	Fe_2_O_3_ (%)	Loss
41.58	28.02	16.03	7.9	2.56	1.61	0.9	0.64	0.37	0.39

**Table 6:** Results of 3d strength test of grouting materials with different amount of cementitious material.

Class	R·SAC 425 (g)	P·O 525 (g)	Fly Ash (g)	Mineral Powder (g)	3d Flexural Strength (MPa)	3d Compressive Strength (MPa)
1	720	90	45	45	8.191	62.43
2	675	90	45	90	8.016	63.53
3	630	90	45	135	7.763	56.82
4	585	90	45	180	7.326	55.34
5	675	90	90	45	7.098	59.48
6	630	90	90	90	7.830	56.89
7	585	90	90	135	7.272	50.54
8	540	90	90	180	6.938	45.82
9	630	90	135	45	7.716	53.70
10	585	90	135	90	6.928	48.35
11	540	90	135	135	6.976	48.49
12	495	90	135	180	6.085	39.57
13	585	90	180	45	6.084	37.80
14	540	90	180	90	7.080	48.22
15	495	90	180	135	6.353	41.09
16	450	90	180	180	6.249	36.79
17	630	180	45	45	6.388	53.86
18	585	180	45	90	6.344	44.99
19	540	180	45	135	6.395	41.33
20	495	180	45	180	5.652	29.72
21	585	180	90	45	6.340	35.18
22	540	180	90	90	6.597	40.36
23	495	180	90	135	5.857	28.75
24	450	180	90	180	4.809	19.39
25	540	180	135	45	6.222	37.46
26	495	180	135	90	5.895	34.71
27	450	180	135	135	4.575	22.37
28	405	180	135	180	4.746	22.03
29	495	180	180	45	4.913	32.66
30	450	180	180	90	3.045	25.62
31	405	180	180	135	3.647	12.55
32	360	180	180	180	2.740	11.05

**Table 7:** Results of workability test of grouting materials with different water cement ratio.

Class	Water Cement Ratio	Polycarboxylate Water Reducing Admixture (%)	Initial Fluidity (mm)	3d Flexural Strength (MPa)	3d Compressive Strength (MPa)
1	0.25	0.3	297	7.188	55.58
2	0.25	0.35	306	8.407	62.24
3	0.25	0.4	317	8.221	61.63
4	0.26	0.3	312	7.249	56.68
5	0.26	0.35	320	8.025	59.95
6	0.26	0.4	324	7.151	55.32
7	0.27	0.3	320	7.915	55.41
8	0.27	0.35	331	6.891	50.92
9	0.27	0.4	341	6.448	46.56
10	0.28	0.3	326	7.125	49.12
11	0.28	0.35	341	6.819	47.83
12	0.28	0.4	350	5.113	42.38

**Table 8:** Effect of retarder on fluidity of grouting materials.

Class	Type of Retarder	Amount of Retarder (%)	Initial Fluidity (mm)	30 min Fluidity (mm)
1	Sodium tetraborate	0.1	299	170
2	Sodium tetraborate	0.2	265	152
3	Sodium tetraborate	0.3	248	140
4	Sodium tetraborate	0.4	220	150
5	Sodium tetraborate	0.5	203	112
6	sodium gluconate	0.05	343	263
7	sodium gluconate	0.06	341	282
8	sodium gluconate	0.07	337	276
9	sodium gluconate	0.08	334	271

**Table 9:** Effect of different defoamers on strength of grouting materials.

Class	Type of Defoamer	Amount of Defoamer (%)	1d Flexural Strength (MPa)	1d Compressive Strength (MPa)
1	silicone defoamer	0.3	6.947	53.71
2	silicone defoamer	0.4	7.277	52.58
3	silicone defoamer	0.5	6.476	50.36
4	silicone defoamer	0.6	6.813	51.75
5	6018	0.3	6.150	52.71
6	6018	0.4	6.553	48.27
7	6018	0.5	6.510	50.54
8	6018	0.6	5.485	32.82
9	H500	0.3	5.393	40.62
10	H500	0.4	6.572	48.26
11	H500	0.5	6.012	44.25
12	H500	0.6	5.154	45.64

**Table 10:** The mixing ratio of grouting materials.

R·SAC (g)	P·O (g)	Fly Ash (g)	Mineral Powder (g)	Quartz Sand (g)	Water (g)	Water Reducing Admixture (g)	Retarder (g)	Defoamer (g)
675	90	45	90	900	225	3.15	0.54	2.7

## Data Availability

Not applicable.
